# Evidence for improved survival with bevacizumab treatment in recurrent high-grade gliomas: a retrospective study with (“pseudo-randomized”) treatment allocation by the health insurance provider

**DOI:** 10.1007/s11060-020-03533-5

**Published:** 2020-05-14

**Authors:** Susanne Hofmann, Manuel Alexander Schmidt, Thomas Weissmann, Ilker Eyüpoglu, Annedore Strnad, Sabine Semrau, Rainer Fietkau, Florian Putz, Sebastian Lettmaier

**Affiliations:** 1 Department of Radiotherapy, Friedrich-Alexander-Universität Erlangen-Nürnberg, Universitaetsstraße 27, 91054 Erlangen, Germany; 2 Department of Neurosurgery, Friedrich-Alexander-Universität Erlangen-Nürnberg, Schwabachanlage 6, 91054 Erlangen, Germany; 3 Department of Neuroradiology, Friedrich-Alexander-Universität Erlangen-Nürnberg, Schwabachanlage 6, 91054 Erlangen, Germany

**Keywords:** Bevacizumab, Recurrent glioma, High-grade glioma, Health insurance, Off-label use, Glioblastoma

## Abstract

**Introduction:**

Despite a large number of trials, the role of bevacizumab (BEV) in the treatment of recurrent high-grade gliomas is still controversial. Evidence regarding an effect on overall survival in this context is ultimately inconclusive. At the Department of Radiation Oncology at Erlangen, Germany we treated a large cohort of patients with recurrent gliomas where bevacizumab use was determined exclusively by the health care provider’s approval of reimbursement.

**Methods:**

61 patients (between 06/2008 and 01/2014) with recurrent high-grade gliomas had reimbursement requests for BEV sent to their health insurance. 37 patients out of 61 (60.7%) had their requests approved and therefore received bevacizumab (BEV-arm) as part of their treatment. The remaining 24 (39.3%) patients received standard therapy without bevacizumab (non-BEV-arm). Survival endpoints were defined with reference to the first BEV request to the health insurance provider.

**Results:**

Median overall survival (OS) for the whole cohort was 7.0 months. OS was significantly better for BEV vs. Non-BEV patients (median, 10.3 vs. 4.2 months, logrank p = 0.023). There was an increased BEV benefit in cases of higher-order recurrences (first order recurrence BEV vs. Non-BEV, 12.5 vs. 10.2 months, p = 0.578) (second or higher order of recurrence, 9.9 vs. 2.6 months, p = 0.010). On multivariate analysis for overall survival the prognostic impact of bevacizumab (HR = 0.43, p = 0.034) remained significant.

**Conclusion:**

Our results suggest an influence of BEV on overall survival in a heavily pretreated patient population suffering from high-grade gliomas with BEV benefit being greatest in case of second or later recurrence.

## Introduction

The long-term prognosis of high-grade gliomas remains poor [1, 2]. Most patients will suffer a tumor recurrence despite receiving gold-standard first-line treatment including surgical tumor removal followed by radiochemotherapy including temozolomide [3, 4]. The options for second-line systemic treatment are unfortunately quite limited. Bevacizumab (BEV)—an agent targeting humoral factors involved in tissue angiogenesis—was first introduced into clinical practice in 2004 following FDA approval and since then has been licensed for the treatment of a broad spectrum of malignant diseases [5].

FDA approval of BEV for recurrent glioblastoma (GBM) was granted in 2009 [6] following the results of the BRAIN-AVF3708 [7] and NCI 06-C-0064E [8] studies which showed a benefit in terms of progression-free (PFS) and overall survival (OS) as compared to historical controls as well as improvements in quality of life due to BEV’s antiedematous and cortisone-sparing effects. Although licensing for gliomas was not granted by the European authorities, off-label use of BEV started becoming widespread in Germany following publication of these results. Neurooncological interest in BEV increased significantly over the following years culminating in the setting-up of trials investigating its role as part of first-line treatment schedules which however, failed to show any improvement in overall survival [9, 10].

This led to a more critical reappraisal of BEV’s role in the recurrent setting. Subsequently, the BELOB trial [11]) provided promising evidence regarding the efficacy of BEV in recurrent high-grade gliomas. However, the phase 3 follow-on trial EORTC-26101 [12] possibly as a result of substantial crossover was not able to confirm the positive results from its smaller precursor revealing no significant benefit in terms of overall survival.

The negative outcome of the EORTC-trial together with a preceding court ruling by the highest court of appeal in Germany dealing with matters of social litigation (Bundessozialgericht; 13.12.2016 [13]) which effectively overturned previous rulings supporting the then wide-spread off-label use of bevacizumab for recurrent high-grade gliomas can be seen as the two milestone events that unfortunately so far have sealed the fate of this substance for treating recurrences of high-grade gliomas in Germany. As a result, bevacizumab currently is unavailable for treatment of recurrent gliomas in Germany with the sole exception that reimbursement requests are occasionally approved in case of radiation necrosis [14].

At the Departments of Radiation Oncology and Neurosurgery of the University Hospital Erlangen, Germany we treated a very large cohort of patients with recurrent high-grade gliomas using bevacizumab in the years prior to its neuro-oncological “demise” in Germany and were thus in a privileged position to obtain a vast amount of first-hand clinical experience of its use for this indication. It was our subjective impression of a marked clinical benefit in several individual patients that led us to retrospectively evaluate the data obtained from these treatments. In this context we realized that the felt randomness of the decision-making process of health care providers in dealing with the reimbursement requests which had been so painful for patients and treating doctors alike might turn out to be of some benefit in retrospectively “proving” a positive effect of bevacizumab on overall survival by providing two cohorts with rather evenly distributed clinical characteristics and without any confounding influences from cross-over between treatment arms upon progression.

## Patients and methods

### Patient population

After publication of the negative first-line results from the AvaGlio [9] and RTOG 0825 [10] trials in 2/2014 treatment requests for BEV were only rarely approved. Therefore, the time span from 6/2008 until 1/2014 was selected for the present analysis. All patients with recurrent high-grade (WHO III and IV) gliomas treated at the Department of Radiation Oncology at Erlangen during this time period were screened for inclusion in this analysis. Patients for whom bevacizumab was recommended were included. The use of bevacizumab was recommended in a total of 61 patients. Because treatment of recurrent gliomas with bevacizumab is “off-label” in the EU approval of reimbursement had to be requested from patients’ health insurance providers prior to treatment initiation in all cases. Approval by insurance providers was obtained in about half of the patients leaving two cohorts of rather similar patient characteristics suggesting a significant random element in the process of decision-making.

For data collection patient health records were reviewed with regard to treatment and survival data as well as correspondence with the patient’s health insurance provider. We evaluated the outcomes of patients in terms of progression-free and overall survival as well as the respective influence of age, ECOG status, WHO grading, additional treatments and insurance status. An investigation of the initial IDH-mutation status was not available [15].

Patients were followed up with clinical visits including adequate imaging (CT, PET-CT, MRI) at 3-monthly intervals for early detection of renewed recurrence.

### Statistics

Progression-free survival and overall survival were calculated from the time of the first BEV request until the time of renewed disease progression or death, respectively. Progression was defined according to the updated Response Assessment in Neurooncology (RANO) criteria for high-grade glioma integrating imaging as well as clinical information [16]. Univariate evaluation of prognostic factors was performed with Kaplan–Meier plots and the log-rank test. Potential prognosticators were also evaluated with univariate and multivariate Cox-regression analysis. A *p*-value < 0.05 was considered statistically significant. SPSS 23 (IBM, Chicago, IL) was used for statistical calculations. Multiplicity adjustments were not performed. *p*-values are therefore descriptive and reflect a Type I error for the individual comparison.

## Results

61 patients fulfilled the criteria for this investigation and were included in the analysis. Patient and tumor characteristics were not significantly different between the BEV and Non-BEV-arm apart from ECOG, which was significantly better in the bevacizumab treated group. A total of 74 reimbursement requests for bevacizumab (BEV) therapy were made and 46 were accepted by the health insurance providers. Besides bevacizumab treatment, a total of 34.4% (21/61) of patients received additional radiotherapy or additional resection. 18.0% (11/61) of all patients received additional resection, 24.6% (15/61) received additional radiotherapy and 8.2% (5/61) received both. (Table 1) In patients with concomitant radiation, most received conventionally-fractionated stereotactic radiotherapy in single doses of 1.8 Gy to a total dose of 45.0 Gy (80.0%, 12/15). The remaining three patients were treated with 10 × 3.0, 5 × 4.0 Gy and 1 × 18 Gy.

**Table 1:** Patient characteristics (n = 61)

Parameter	All patients (n = 61)	Non-BEV (n = 24)	BEV (n = 37)	p for difference
Age at reimbursement request				0.653^a^
Median (range)	57 years (20–71)	59 years (20–71)	57 years (25–70)	
Gender, n (%)				0.171^b^
Male	40 (65.6%)	13 (54.2%)	27 (73.0%)	
Female	21 (34.4%)	11 (45.8%)	10 (27.0%)	
WHO grade				1.000^b^
Grade III	11 (18.0%)	4 (16.7%)	7 (18.9%)	
Grade IV	50 (82.0%)	20 (83.3%)	30 (81.1%)	
ECOG				0.001^b^
ECOG 0	5 (8.2%)	–	5 (13.5%)	
ECOG 1	26 (42.6%)	6 (25.0%)	20 (54.1%)	
ECOG 2	17 (27.9%)	11 (45.8%)	6 (16.2%)	
ECOG 3	6 (9.8%)	1 (4.2%)	5 (13.5%)	
No information	7 (11.5%)	6 (25.0%)	1 (2.7%)	
MGMT status				0.524^b^
Negative	13 (21.3%)	4 (16.7%)	9 (24.3%)	
Positive	5 (8.2%)	1 (4.2%)	4 (10.8%)	
No information	43 (70.5%)	19 (79.2%)	24 (64.9%)	
Additional resection				0.502^b^
Yes	11 (18.0%)	3 (12.5%)	8 (21.6%)	
No	50 (82.0%)	21 (87.5%)	29 (78.4%)	
Additional radiotherapy				0.127^b^
Yes	15 (24.6%)	3 (12.5%)	12 (32.4%)	
No	46 (75.4%)	21 (87.5%)	25 (67.6%)	
nth recurrences				0.637^a^
Median (range)	2 (1–4)	2 (1–3)	2 (1–4)	
nth recurrences, n (%)				0.883^b^
1st recurrence	21 (34.4%)	8 (33.3%)	13 (35.1%)	
2nd recurrence	32 (52.5%)	14 (58.3%)	18 (48.6%)	
3rd recurrence	7 (11.5%)	2 (8.3%)	5 (13.5%)	
4th recurrence	1 (1.6%)	0 (0.0%)	1 (2.7%)	

^a^Two-sample T-test

^b^Fisher’s exact test

The median time interval from the date of the reimbursement request to receiving approval of reimbursement was 29 days. The median interval from diagnosis of recurrence until initiation of bevacizumab treatment was 54 days. In comparison the median interval from diagnosis of recurrence to initiation of standard treatment for patients not receiving bevacizumab was 30 days. A total of 37 patients received bevacizumab treatment, 5 of those received bevacizumab for more than one recurrence. In 25 patients bevacizumab was given in combination with chemotherapy, either irinotecan (n = 24) or CCNU (n = 1). For four patients no data were available as to whether bevacizumab was given as mono-therapy or in combination. A median of 8 doses of bevacizumab were administered per patient.

**Fig. 1:**
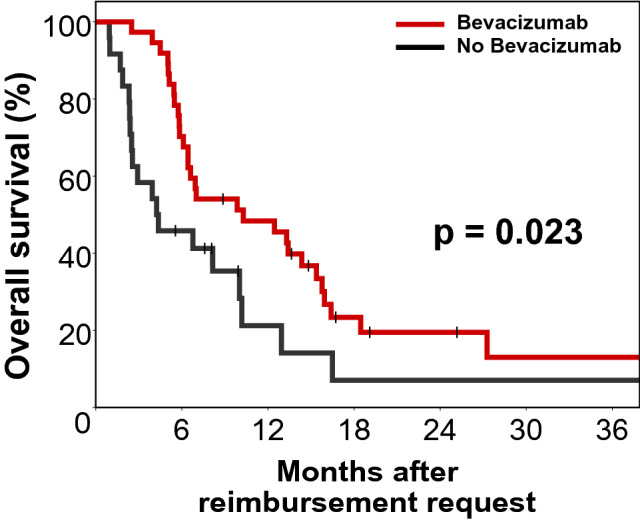
Kaplan–Meier plot for Overall survival (n = 61). Median overall survival for the BEV-arm (n = 37) vs. Non-BEV-arm (n = 24) was 10.3 vs. 4.2 months (log rank p = 0.023)

After a median follow-up time of 25.2 months, 78.7% of patients (n = 48) had died. The median overall survival for the whole cohort was 7.0 months following the first BEV request. The median OS in the BEV group (n = 37) was significantly longer at 10.3 months (p = 0.023) than in the Non-BEV group (n = 24) which was 4.2 months. (Fig. 1) When comparing the BEV-mono group with the group receiving BEV as combination therapy there was no significant OS benefit (p = 0.681).

There was an increased BEV benefit for patient with higher-order recurrences. While patients with first glioma recurrence showed only a small, non-significant improvement (n = 21, BEV vs. Non-BEV, median OS 12.5 vs. 10.2 months, p = 0.578), patients with higher-order recurrences showed a marked improvement, which was highly significant (second or higher order of recurrence n = 40, median OS 9.9 vs. 2.6 months, p = 0.010).

With the reimbursement decision of health care providers being instrumental in assigning a patient’s treatment we also analyzed overall survival with respect to a patient’ s insurance status. Out of 48 non-privately insured patients, 24 (50.0%) received BEV, whereas all 13 (100%) privately insured patients were granted BEV treatment. However, there was no significant difference in survival between privately insured vs. non-privately insured patients (median OS, 6.4 vs. 8.2 months, p = 0.355).

**Fig. 2:**
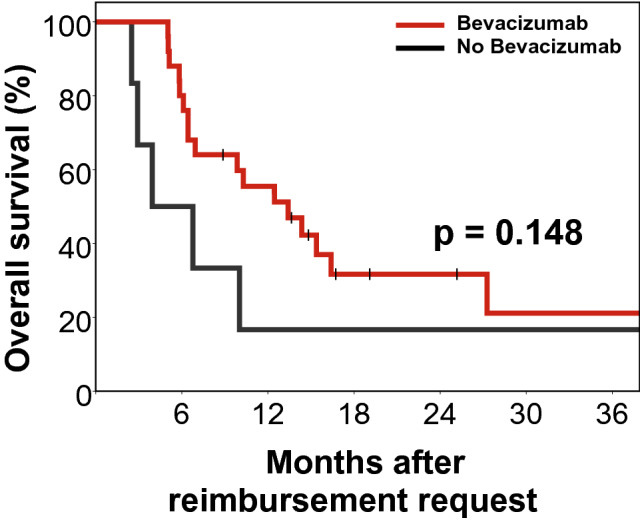
Kaplan–Meier plot for Overall survival in the ECOG 0–1 subgroup (n = 31)

As both treatment groups differed in terms of the distribution of the ECOG status, we assessed the prognostic impact of BEV treatment again in the subgroup of patients with a favorable ECOG status of 0–1. In total, 31 patients were either ECOG 0 or ECOG 1 at study entry. Of these patients, 25 (80.6%) received BEV and 6 (19.4%) did not. Interestingly, although as expected significance was lost due to the small number of patients, median survival was substantially higher in the BEV group (13.4 vs. 3.9 months, p = 0.148) and the Kaplan–Meier plot also was consistent with improved survival in the BEV treated patients (Fig. 2).

As the study period extended from 2008 to 2014, an additional possible confounder was the time of study entry. However, when the date of the first reimbursement request, which marked the study entry, was dichotomized at the median no significant effect on OS was found in univariate Cox’s regression analysis (HR 0.84 for later entry, p = 0.562). In addition, probability of BEV approval was not significantly higher for later entry dates, where treatment could have improved (Median date of study entry, 07.09.2010 vs. 13.08.2010 for BEV vs. no BEV treatment, Wilcoxon rank-sum p = 0.408). Similarly, when included among the other prognosticators in multivariate analysis, the date of study entry was no significant prognostic factor (HR 0.81, p = 0.524).

**Fig. 3:**
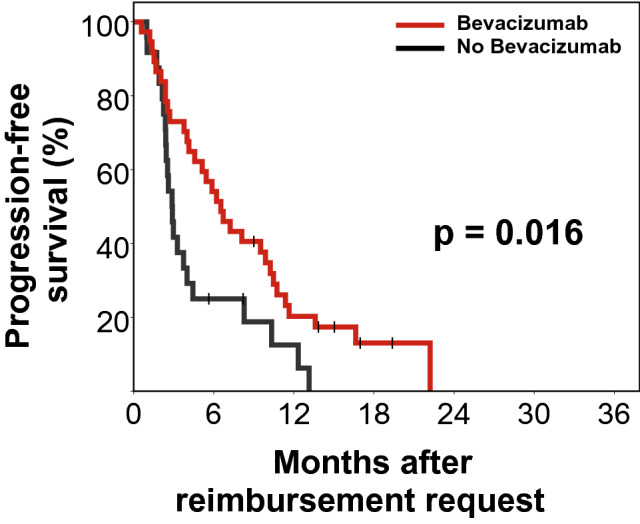
Kaplan–Meier plot for progression-free survival (n = 61). Median progression-free survival for the BEV-arm (n = 37) vs. Non-BEV-arm (n = 24) was 6.5 vs. 2.9 months (log rank p = 0.016). Progression was defined according to the updated RANO criteria

Progression-free survival (PFS) was significantly improved for patients that received BEV in comparison to patients that did not (median PFS, 6.5 months vs. 2.9 months and 6-months PFS, 54.1% vs. 25.0%, p = 0.016, Fig. 3). As with OS, PFS benefit for the BEV group was not significant at first recurrence (median PFS, 6.7 vs. 3.7 months, 6-months PFS 61.5% vs. 50.0%, p = 0.562) but was more pronounced and significant, if BEV was requested at subsequent recurrences (median PFS, 5.9 vs. 2.6 months, 6-months PFS, 50.0% vs. 12.5%, p = 0.023). In the bevacizumab group, 35.1% (13/37) of patients continued treatment beyond progression for a median of 3.0 months (range 0.4–7.6 months). Additional resection and additional radiotherapy showed a reduced hazard ratio for progression or death but failed to reach significance (PFS, HR = 0.60, p = 0.179 and HR = 0.54, p = 0.073 respectively).

### Multivariate analysis

In multivariate analysis for OS the prognostic impact of bevacizumab (HR = 0.43, p = 0.034) remained significant when accounting for ECOG, age, WHO grade, additional treatments, insurance type and the number of recurrences before planned initiation of bevacizumab. Higher orders of recurrence and higher WHO grade were associated with a significantly increased risk of death with hazard ratios of 1.72 and 2.79, respectively. Age, ECOG, resection or radiotherapy for recurrence and the type of insurance did not have a significant influence on OS in multivariate analysis (Table 2).

**Table 2:** Univariate and multivariate Cox’s regression analysis of prognostic factors for overall survival (n = 61)

Parameter	Univariate		Multivariate	
	HR (95% CI)	*p*-value	HR (95% CI)	*p*-value
Bevacizumab, yes vs. no	**0.51 (0.28**–**0.92)**	**0.026**	**0.43 (0.20**–**0.94)**	**0.034**
WHO grade, °IV vs. °III	**1.93 (0.86**–**4.31)**	**0.110**	**2.79 (1.08**–**7.18)**	**0.034**
nth recurrence	**1.11 (0.75**–**1.63)**	**0.603**	**1.72 (1.04**–**2.84)**	**0.033**
ECOG, per point	1.21 (1.01–1.44)	0.038	1.22 (0.99–1.51)	0.062
Age, per year	1.02 (0.99–1.05)	0.132	1.02 (0.99–1.05)	0.155
Resection, yes vs. no	0.58 (0.26–1.30)	0.186	0.97 (0.38–2.45)	0.941
Radiotherapy, yes vs. no	0.64 (0.32–1.30)	0.217	0.63 (0.29–1.36)	0.237
Insurance, private vs. non-private	1.10 (0.56–2.17)	0.782	1.89 (0.82–4.34)	0.135

Bold values indicate significant prognostic factors in multivariate analysis

## Discussion

In this retrospective study with a total of 61 patients we were able to detect significant differences in OS (median, 10.3 vs. 4.2 months) and PFS (median, 6.5 vs. 2.9 months) favoring the BEV treated cohort. When accounting for multiple prognostic factors on multivariate analysis the prognostic effect of bevacizumab treatment on overall survival remained significant. While the conclusions drawn from our data suffer from the obvious limitation of not being based on a truly randomized data set, a true effect of bevacizumab remains a likely explanation of the large and statistically significant differences in survival parameters seen between the BEV and non-BEV arms.

This interpretation would seem less precarious if it was not in seeming contradiction with findings from large-scale randomized trials that failed to show a survival benefit in both the primary and recurrent treatment settings [9, 10, 12]. The interpretation of our data as a direct survival benefit conferred by BEV will only stand if this conflict can be resolved.

When trying to come to such a resolution the first point to consider is the extent to which the trials that so far have provided contradictory “answers” have actually addressed the same scientific “question”. Both AVAglio [9] and RTOG 0825 [10], two large randomized trials that investigated the role of BEV in the first-line, evaluated the addition of bevacizumab to the standard Stupp schedule [3] at first diagnosis and failed to show a significant improvement in overall survival. While it is obvious that that improvement of overall survival in the first-line situation gives a strong indication for a substance’s efficacy also for recurrences the reverse of this statement need not necessarily be true. Consequently, the failure of those trials to improve overall survival just proves a lack of benefit of adding BEV at that particular stage in a patient’s course of treatment and does not exclude a potential benefit further down the line where treatment alternatives become scarcer.

When trying to find possible explanations for the discrepancy of our findings with those of EORTC 26101, the largest trial so far investigating the role of BEV in the second line treatment of high-grade gliomas, it is important to look more closely at patient characteristics and the way patients were treated [12]. In the EORTC trial only patients with first recurrences after standard treatment were included and for ethical reasons salvage treatment with bevacizumab was allowed if patients progressed on CCNU alone. Despite a marked and significant improvement in progression- free survival (4.2 vs. 1.5 months in favor of BEV + CCNU vs. CCNU monotherapy) no significant improvement in overall survival was seen on intention-to-treat analysis. Of note, however, 35.5% of the patients randomized to CCNU monotherapy were switched to BEV during the course of their treatment and the trial therefore effectively addressed the question of whether adding BEV to CCNU up-front in patients with first recurrence was superior to allowing it to be added only at the time of progression on CCNU as far as its effect on overall survival is concerned. In our study on the other hand most patients had BEV treatment for second or higher order of recurrence. Also, sadly, patients in the non-BEV group whose tumor showed progression, did not have the option to receive salvage treatment with bevacizumab due to lack of funding. The question our study would be entitled to answer, therefore, is whether excluding BEV entirely from treatment of recurrent gliomas of any order may worsen overall survival. In this sense, there is no contradiction between our findings and those of the large randomized trials on the subject since the questions addressed are different. Interestingly, PFS in these trials was comparable to that seen in the present study.

Looking further at data from the literature the lack of a relevant cross-over phenomenon may also explain the positive outcome of the BELOB trial that investigated the addition of BEV to CCNU with a three-armed design but was not confounded by crossover from the CCNU arm to the BEV containing arms. There was a significant difference in OS in this trial with a median OS of 12 months in the CCNU + BEV arm as compared to 8 months in the arm receiving CCNU monotherapy [11].

Finally, data from a recently published German study that retrospectively evaluated the role of BEV for recurrent glioblastomas as last-line treatment and in particular following progression on CCNU provided strong evidence in favor of BEV in this context [17]. Interestingly, the authors were also able to find a correlation between radiological response and overall survival with 21.3% of radiological responders being alive at 12 months as opposed to 0% for non-responders. This is noteworthy since it suggests that radiological improvement may be more than just a “cosmetic” effect attributable to BEVs direct effects on vascular permeability masking actual tumor progression through a phenomenon known as “pseudoresponse” which has frequently been invoked when trying to explain the vast discrepancy between PFS and OS found in most if not all relevant trials.

## Conclusion

Our data provide evidence in favor of BEV conferring an overall survival benefit in recurrent high-grade glioma. Benefit from BEV treatment was most pronounced in second or later recurrence, while it did not reach significance in the subgroup with first recurrence. The fact that BEV treatment was completely dependent on approval by insurance providers precluded any cross-over effects. Our study adds to the evidence that BEV might improve survival in multiple recurrent high-grade gliomas. We hope that our data combined with a critical reappraisal of high quality data available in the literature would help end the ongoing blockade to fund bevacizumab treatment for multiple recurrent high-grade gliomas in EU countries and lead licensing authorities to follow the example of the US where existing evidence was in itself deemed sufficient to warrant licensing for this important indication.

## Data Availability

Data is available upon request. However legal restrictions especially the EU General Data Protection Regulation (GDPR), the German Data Protection Laws and the Bavarian Hospital law apply, so that some requests may have to be declined partially or completely. Not applicable.

## References

[1] Kaatsch P SC, Katalinic A, Hentschel S, Wolf U et al (2015) Krebs in Deutschland 2011/2012. 10. Ausgabe. Robert Koch-Institut (Hrsg)und die Gesellschaft der epidemiologischen Krebsregister in Deutschland e.V. (Hrsg), Berlin. 10.17886/rkipubl-2015-004

[2] Ostrom QT, Gittleman H, Liao P, Rouse C, Chen Y, Dowling J, Wolinsky Y, Kruchko C, Barnholtz-Sloan J (2014). CBTRUS statistical report: primary brain and central nervous system tumors diagnosed in the United States in 2007–2011. Neuro-oncology.

[3] Stupp R, Mason WP, van den Bent MJ, Weller M, Fisher B, Taphoorn MJ, Belanger K, Brandes AA, Marosi C, Bogdahn U, Curschmann J, Janzer RC, Ludwin SK, Gorlia T, Allgeier A, Lacombe D, Cairncross JG, Eisenhauer E, Mirimanoff RO (2005). Radiotherapy plus concomitant and adjuvant temozolomide for glioblastoma. N Engl J Med.

[4] Hart MG, Garside R, Rogers G, Stein K, Grant R (2013). Temozolomide for high grade glioma. Cochrane Database Syst Rev.

[5] Krebsgesellschaft (2018) Bevacizumab. https://www.krebsgesellschaft.de/onko-internetportal/basis-informationen-krebs/basis-informationen-krebs-allgemeine-informationen/wirkstoff-glossar/bevacizumab.html

[6] Cohen MH, Shen YL, Keegan P, Pazdur R (2009). FDA drug approval summary: bevacizumab (Avastin) as treatment of recurrent glioblastoma multiforme. Oncologist.

[7] Friedman HS, Prados MD, Wen PY, Mikkelsen T, Schiff D, Abrey LE, Yung WK, Paleologos N, Nicholas MK, Jensen R, Vredenburgh J, Huang J, Zheng M, Cloughesy T (2009). Bevacizumab alone and in combination with irinotecan in recurrent glioblastoma. J Clin Oncol.

[8] Kreisl TN, Kim L, Moore K, Duic P, Royce C, Stroud I, Garren N, Mackey M, Butman JA, Camphausen K, Park J, Albert PS, Fine HA (2009). Phase II trial of single-agent bevacizumab followed by bevacizumab plus irinotecan at tumor progression in recurrent glioblastoma. J Clin Oncol.

[9] Chinot OL, Wick W, Mason W, Henriksson R, Saran F, Nishikawa R, Carpentier AF, Hoang-Xuan K, Kavan P, Cernea D, Brandes AA, Hilton M, Abrey L, Cloughesy T (2014). Bevacizumab plus radiotherapy-temozolomide for newly diagnosed glioblastoma. N Engl J Med.

[10] Gilbert MR, Dignam JJ, Armstrong TS, Wefel JS, Blumenthal DT, Vogelbaum MA, Colman H, Chakravarti A, Pugh S, Won M, Jeraj R, Brown PD, Jaeckle KA, Schiff D, Stieber VW, Brachman DG, Werner-Wasik M, Tremont-Lukats IW, Sulman EP, Aldape KD, Curran WJ, Mehta MP (2014). A randomized trial of bevacizumab for newly diagnosed glioblastoma. N Engl J Med.

[11] Taal W, Oosterkamp HM, Walenkamp AME, Dubbink HJ, Beerepoot LV, Hanse MCJ, Buter J, Honkoop AH, Boerman D, de Vos FYF, Dinjens WNM, Enting RH, Taphoorn MJB, van den Berkmortel FWPJ, Jansen RLH, Brandsma D, Bromberg JEC, van Heuvel I, Vernhout RM, van der Holt B, van den Bent MJ (2014). Single-agent bevacizumab or lomustine versus a combination of bevacizumab plus lomustine in patients with recurrent glioblastoma (BELOB trial): a randomised controlled phase 2 trial. Lancet Oncol.

[12] Wick W BA, Gorlia T, Bendszus M, Sahm F, Bent V B et al (2016) EORTC 26101 phase III trial exploring the combination of bevacizumab and lomustine in patients with first progression of a glioblastoma. J Clin Oncol 34

[13] Jurion (2016) Bundessozialgericht Urt. v. 13.12.2016, Az.: B 1 KR 10/16 R,Keine Kostenerstattung der gesetzlichen Krankenversicherung für die Behandlung eines rezidivierenden Glioblastoms mit dem Fertigarzneimittel Avastin. https://www.jurion.de/urteile/bsg/2016-12-13/b-1-kr-10_16-r/. Accessed 25 Apr 2019

[14] Bodensohn R, Hadi I, Fleischmann DF, Corradini S, Thon N, Rauch J, Belka C, Niyazi M (2020). Bevacizumab as a treatment option for radiation necrosis after cranial radiation therapy: a retrospective monocentric analysis. Strahlenther Onkol.

[15] Back M, Jayamanne D, Brazier D, Newey A, Bailey D, Schembri G, Hsiao E, Khasraw M, Wong M, Kastelan M, Brown C, Wheeler H (2019). Pattern of failure in anaplastic glioma patients with an IDH1/2 mutation. Strahlenther Onkol.

[16] Wen PY, Macdonald DR, Reardon DA, Cloughesy TF, Sorensen AG, Galanis E, Degroot J, Wick W, Gilbert MR, Lassman AB, Tsien C, Mikkelsen T, Wong ET, Chamberlain MC, Stupp R, Lamborn KR, Vogelbaum MA, van den Bent MJ, Chang SM (2010). Updated response assessment criteria for high-grade gliomas: response assessment in neuro-oncology working group. J Clin oncol.

[17] Wenger KJ, Wagner M, You SJ, Franz K, Harter PN, Burger MC, Voss M, Ronellenfitsch MW, Fokas E, Steinbach JP, Bahr O (2017). Bevacizumab as a last-line treatment for glioblastoma following failure of radiotherapy, temozolomide and lomustine. Oncol Lett.

